# Structural organization of the neuronal pathways of the superior ovarian nerve in the rat

**DOI:** 10.1186/s13048-023-01109-1

**Published:** 2023-01-27

**Authors:** Cesar F. Pastelin, María E. Rivera-Castro, Nancy Mirto-Aguilar, Carolina Moran

**Affiliations:** 1 Facultad de Medicina Veterinaria y Zootecnia, Benemérita Universidad Autónoma de Puebla, Puebla, México; 2 Centro de Investigación en Fisicoquímica de Materiales, Instituto de Ciencias, Benemérita Universidad Autónoma de Puebla, Puebla, México; 3 Doctorado en Investigaciones Cerebrales, Instituto de Investigaciones Cerebrales, Universidad Veracruzana, Xalapa, Veracruz, México

**Keywords:** Superior ovarian nerve, Suprarenal ganglion, Celiac ganglion, Suspensory ligament, Ovary

## Abstract

**Background:**

In the rat, studies have shown that ovary innervation arrives via the superior ovarian nerve (SON) and the ovarian plexus nerve, which originates from the celiac plexus (CP). In the present study, we performed a neuroanatomical technique to investigate the anatomy of the SON between the ovary and the CP.

**Results:**

We found that the SON fibers were concentrated on the lateral border of the suprarenal ganglion and projected towards, then inserted into the suspensory ligament. Then, it ran parallel to the long axis of the ligament to reach and innervate the ovaries. At this level, the SON was composed of two coiled nerve fibers, each between 10 and 15 µm in diameter. The SON was linked to three different ganglia: the suprarenal ganglia, the celiac ganglia, and the superior mesenteric ganglion.

**Conclusions:**

The postganglionic fibers that project to the ovary via the SON emerge from the suprarenal ganglia. The trajectories on the right and left sides to each ovary are similar. The somas of ipsilateral and contralateral SON neurons are located in the prevertebral ganglia, mostly in the celiac ganglia.

## Background

It is known that, in the rat, the ovarian suspensory ligament (cranial suspensory ligament) is a fibromuscular, cord-like structure that extends cranially, from the hilum of the ovary to the lower edge of the thoracic cage. At the rostral end, the muscle has a fan-shaped structure that attaches to the lower-most rib [1–4]. Additionally, blood vessels and adrenergic innervation run along the wall of the suspensory ligament [5, 6]. Because it is one of the main routes to innervate the ovary, this innervation is called the superior ovarian nerve (SON) [7]. Baljet and Drukker [8] found that postganglionic SON fibers ran alongside the suspensory ligament and entered the hilum of the ovary; then, they were distributed around the ovarian stroma and follicles. Subsequently, other studies found that SON postganglionic fibers were embedded in smooth muscle and ran parallel to the long axis of the suspensory ligament [1, 9].

In the rat, the ovarian nerves are derived from the celiac plexus (CP), the intermesenteric plexus, and the superior lumbar splanchnic nerves. Baljet and Drukker [8] showed that the contributions from the suprarenal ganglion (SG) of the CP were bundles of nerve fibers that ran in the direction of the suspensory ligament of the ovary. Later, our laboratory and others studied these fibers with retrograde neuronal tracer applied directly to the SON (i.e., the SON was cut and immersed in True Blue solution) or injected into the ovarian bursa. Those studies showed that sympathetic postganglionic somas were located in the celiac superior mesenteric ganglia complex (CSMG) [10, 11]. On the other hand, other studies showed that the postganglionic somas that gave rise to the fibers that projected to the ovary through the SON were located mainly in the celiac ganglion (CG) [12, 13].

The ovarian nerves are involved in various pathological conditions of the ovary. Therefore, it is important to know precisely where to perform surgical procedures in the SON. Our laboratory aimed to elaborate a detailed route of ovarian innervation. Considering its asymmetric characteristics, we started with the ovarian plexus nerve (OPN) [14]. From that study, it remained unclear whether the SON ran lateral to the suspensory ligament or was immersed in the suspensory ligament. In the present study, we aimed to corroborate the pathway and identify the origin of the SON in the female rat.

## Methods

### Animals

The experimental protocol for the use of rats was evaluated and approved by the Committee for the Care and Use of Laboratory Animals, at Benemérita Universidad Autónoma de Puebla (100–310-955-UALVIEP-19/2). Technical specifications related to the production, care, and use of animals are specified in the guidelines of the Mexican Council on Laboratory Animal Care (NOM-062-Z00-1999).

### Experimental design

Three-month-old, virgin rats of the CIIZ-V strain (250–350 g body weight) were studied. The rats were maintained on a 12/12-h light/dark cycle with food (Lab-Diet 5001, Rodent diet) and water, provided ad libitum. Estrous cycles were monitored with daily vaginal smears. Only rats that showed at least two consecutive four-day cycles were used in the experiment.

Rats were randomly assigned to one of the following bilateral studies: Gross anatomy (*n* = 10), acetylcholinesterase histochemistry (*n* = 4), or scanning electron microscopy (SEM; *n* = 4). Additionally, rats were analyzed unilaterally (right side) with a fluorescent retrograde tracer (*n* = 4).

Surgery was performed under anesthesia with ketamine (90 mg/kg) and xylazine (15 mg/kg) at 9:00–10:00 am, on diestrus. All rats were sacrificed with an overdose of sodium pentobarbital (60 mg/kg, i.p.; Anestesal, Smith Kline, Mexico City, Mexico).

### Gross anatomy

Rats were anesthetized with an intraperitoneal injection of urethane (ethyl carbamate, 1.2 g/kg; Sigma-Aldrich, St. Louis, MO). To expose the SON, a 3-cm paramedian skin incision was made. The SON was located and dissected. Images viewed through a stereo microscope (LEICA M80, GERMANY) were captured in hand drawings. Additionally, digital photographs were acquired with a digital camera (PROGRES GRYPHAX® SUBRA, Germany) and managed in PROGRES GRYPHAX® Microscope Camera Software.

### Acetylcholinesterase histochemistry

The acetylcholinesterase histochemistry is a technique useful in the diagnosis, gross anatomy and recognition of nervous fibers of the peripheral nervous system [8, 15–17]. In this work it is used it to localize very thin fibers.

Rats were sacrificed with an overdose of sodium pentobarbital, then were perfused transcardially with 300 ml saline solution (0.9% NaCl). The SON was dissected from the uterus to the diaphragm, and the dissection included the ovaries, kidneys, and the suspensory ligament of the ovary. A longitudinal incision was made in the abdominal-pelvic regions to extend it flat, and fat tissue was removed. Thereafter, the tissue was fixed by immersing in 10% formalin for at least 48 h. The tissue was washed in phosphate buffer (PBS) 1X, then incubated for 2.5–3 h at room temperature in a solution of acetylthiocholine-iodide dissolved in 0.1 M sodium-hydrogen-maleate, 0.1 M sodium-citrate, 30 mM CuSO4, 5 mM potassium-ferricyanide, and 0.1% Triton X-100 (pH 6.0). Next, the tissue was, dehydrated with ascending alcohol concentrations, cleared with xylene, and observed and photographed under a stereo microscope (LEICA M80, GERMANY). Digital photographs were taken with digital camera (PROGRES GRYPHAX® SUBRA, Germany).

### Electron microscopy

The micromorphology of the suspensory ligament of the ovary was observed and analyzed with a Tescan Vega SEM (TS 51365B, Tescan, Brno, Czech Republic). Briefly, the suspensory ligament of the ovary tissue was fixed in a 4% glutaraldehyde solution for 24 h and washed with PBS (pH = 7.1). The tissue was then dehydrated in a series of ethanol solutions (30%, 50%, 70%, 85%, 96%, and absolute ethanol). Subsequently, the tissue was dried with liquid CO2 for 10 min in a Samdri-795 point dryer (Tousimis, Rockville, MD), incorporated into aluminum pin stubs, and sputter-coated with a 10-nm gold layer, with a Vacuum Desk II sputter coater (Denton Vacuum, LLC, Moorestown, NJ). The scanning was performed with an accelerating voltage of 20 kV.

### Fluorescent retrograde tracer

Four rats in proestrus were anesthetized with an intraperitoneal injection of ketamine (90 mg/kg) and xylazine (15 mg/kg, i.p.). Surgery was performed with a unilateral incision, starting at 3 cm below the lower-most rib, under a stereomicroscope (LEICA M80, GERMANY). The right OPN was surgically denervated, and the right SON remained intact. The right ovarian bursa was then injected with 5 µl of True Blue tracer (TB, diluted 1% with distilled water). The ovary was carefully cleaned, dried, and returned to the abdominal cavity. The rats were maintained in a warm chamber until they recovered from anesthesia, and they were given antibiotics and analgesia. Then, the rats were returned to the bioterium for maintenance.

Seven days later, the rats were anesthetized and sacrificed by performing transcardial perfusion with 200 mL cold saline solution, followed by 200 mL fixative solution (4% paraformaldehyde in PBS at pH 7.1). After perfusion, the CG, SG, and superior mesenteric ganglion (SMG) were stored in the fixative solution (approximately 2 h). The nervous tissue was cryoprotected by immersing in a series of sucrose solutions (10%, 20%, and 30% sucrose in PBS 1X); the tissue was stored for 24–48 h at 4 °C in each solution. Next, the tissue was embedded in Tissue-Tek medium for frozen tissue samples (Sakura Finetek USA, Torrance, CA) and sectioned in 20-µm slices with a cryostat (77,210,163, Thermo Scientific, Waltham, MA). The sections were mounted in poly-L-lysine (adhesive slide solution, Sigma-Aldrich, St. Louis, MO) and placed on clean, glass microscope slides.

The sections were analyzed with epifluorescence microscopy (Olympus BX 41, Olympus Corporation, Tokyo, Japan). TB-positive neurons were observed with UV light (340–380 nm excitation filters). The raw number of labeled neurons was adjusted with the Abercrombie´s correction factor. The sections were photographed with a digital camera (Evolutions VF, Media Cybernetics, Canada). Digital photomicrographs were saved as TIFF files, and images were analyzed and measured with Image-Pro Premier 9.2 software (Media Cybernetics, Bethesda, MD), after contrast and brightness were automatically adjusted. The number of neurons labeled with TB in the CG, the SG, and the SMG were counted, and results are presented as the mean ± standard error of the mean for the four rats.

## Results

### Anatomic description of the SON

All animals analyzed showed the same anatomical organization at the SON origin. The right and left sides of the SON displayed a similar trajectory, from the SG to the ipsilateral ovary, which ruled out a direct anatomic connection to the CG.

**Fig. 1:**
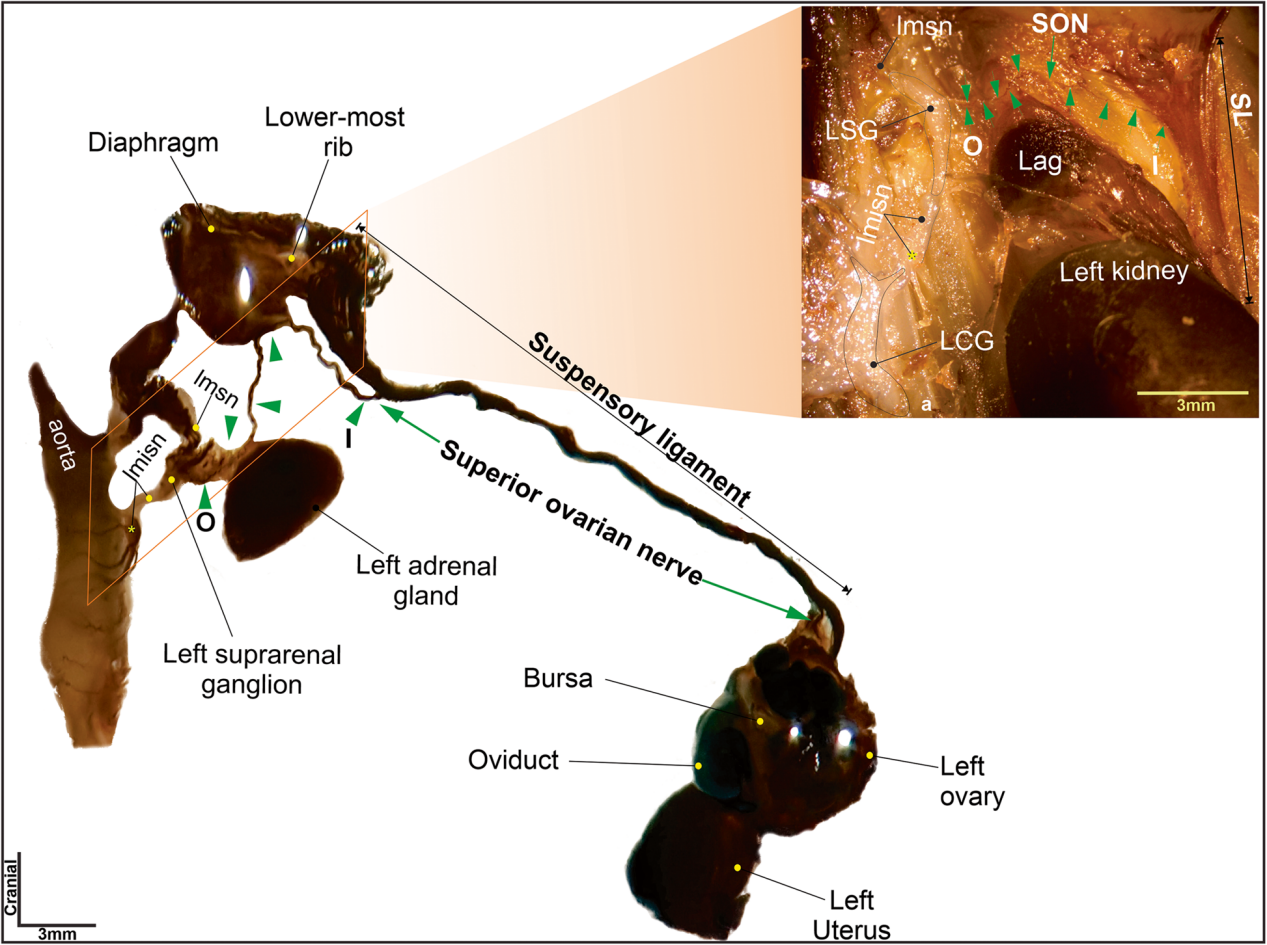
Photographs of abdominal tissues treated with acetylcholinesterase histochemistry show the course of the superior ovarian nerve (green arrows) and its relationship to the adrenal gland, lower-most rib, suspensory ligament (SL), kidney, and ovary in the female rat. SON, superior ovarian nerve; LSG, left suprarenal ganglion, Lag, left adrenal gland; lmsn, left major splanchnic nerve; lmisn, left minor splanchnic nerve; O, origin of SON; I, insertion of SON to the suspensory ligament; LCG, left celiac ganglion

**Fig. 2:**
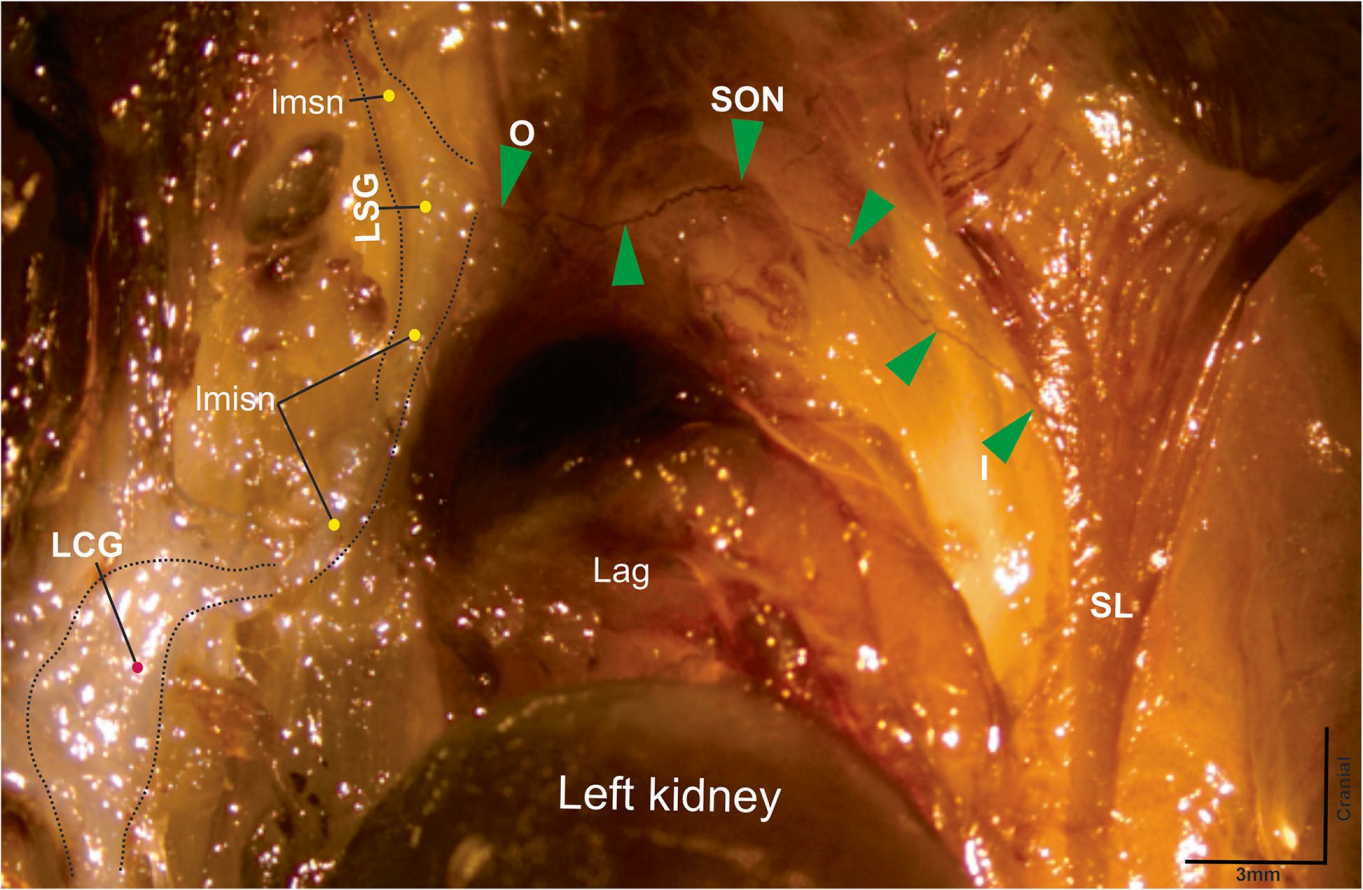
Photograph of the tissue between the left kidney and the diaphragm, treated with acetylcholinesterase histochemistry. Green arrows show the course of the superior ovarian nerve and its relationship to the left adrenal gland (Lag). SL, suspensory ligament; SON, superior ovarian nerve; LSG, left suprarenal ganglion; LCG, left celiac ganglion; lmsn, left major splanchnic nerve; lmisn, left minor splanchnic nerve; O, origin of the SON; I, insertion of the SON to the suspensory ligament

**Fig. 3:**
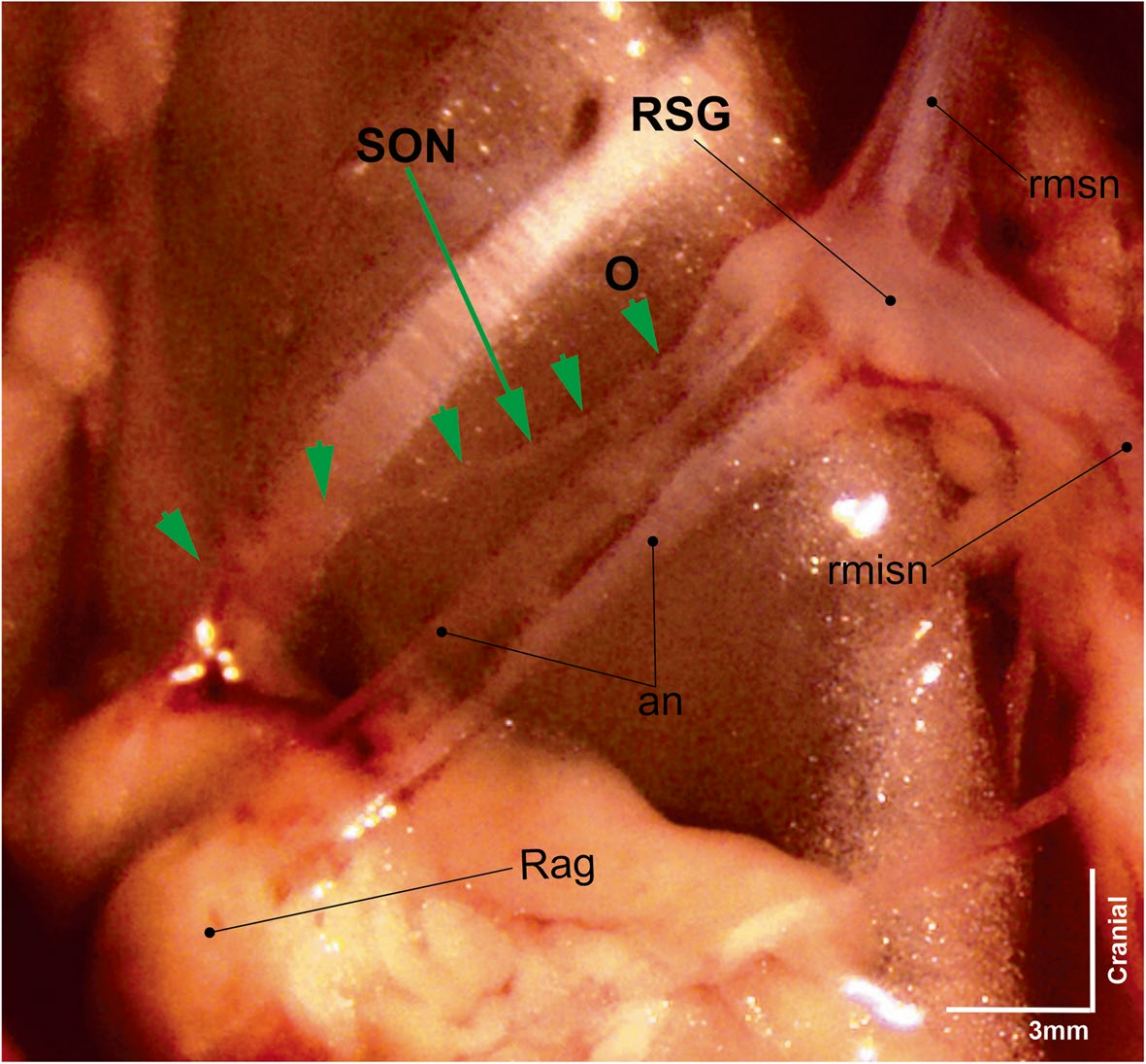
Photograph of the gross anatomy of the origin of the right superior ovarian nerve. SON, superior ovarian nerve; O, origin of the SON; RSG, right suprarenal ganglion; Rag, right adrenal gland; rmsn, right major splanchnic nerve; rmisn, right minor splanchnic nerve; an, adrenal nerves

The postganglionic fibers of the SON emerged from the lateral border of the SG (Figs. 1, 2 and 3) and ran freely over the lateral border of the adrenal gland. Then, they traveled to the area of insertion at the suspensory ligament (Figs. 1, 2, and 5). A few millimeters before reaching the area of insertion, below the diaphragm, these fibers traveled caudally and inserted into the suspensory ligament (Figs. 1 and 2), where they continued until they innervated the ipsilateral ovary (Fig. 5). The total length of the SON, from the SG to the ovary was 17.8 ± 0.3 mm, and no branches were observed (Figs. 1 and 2).

**Fig. 4:**
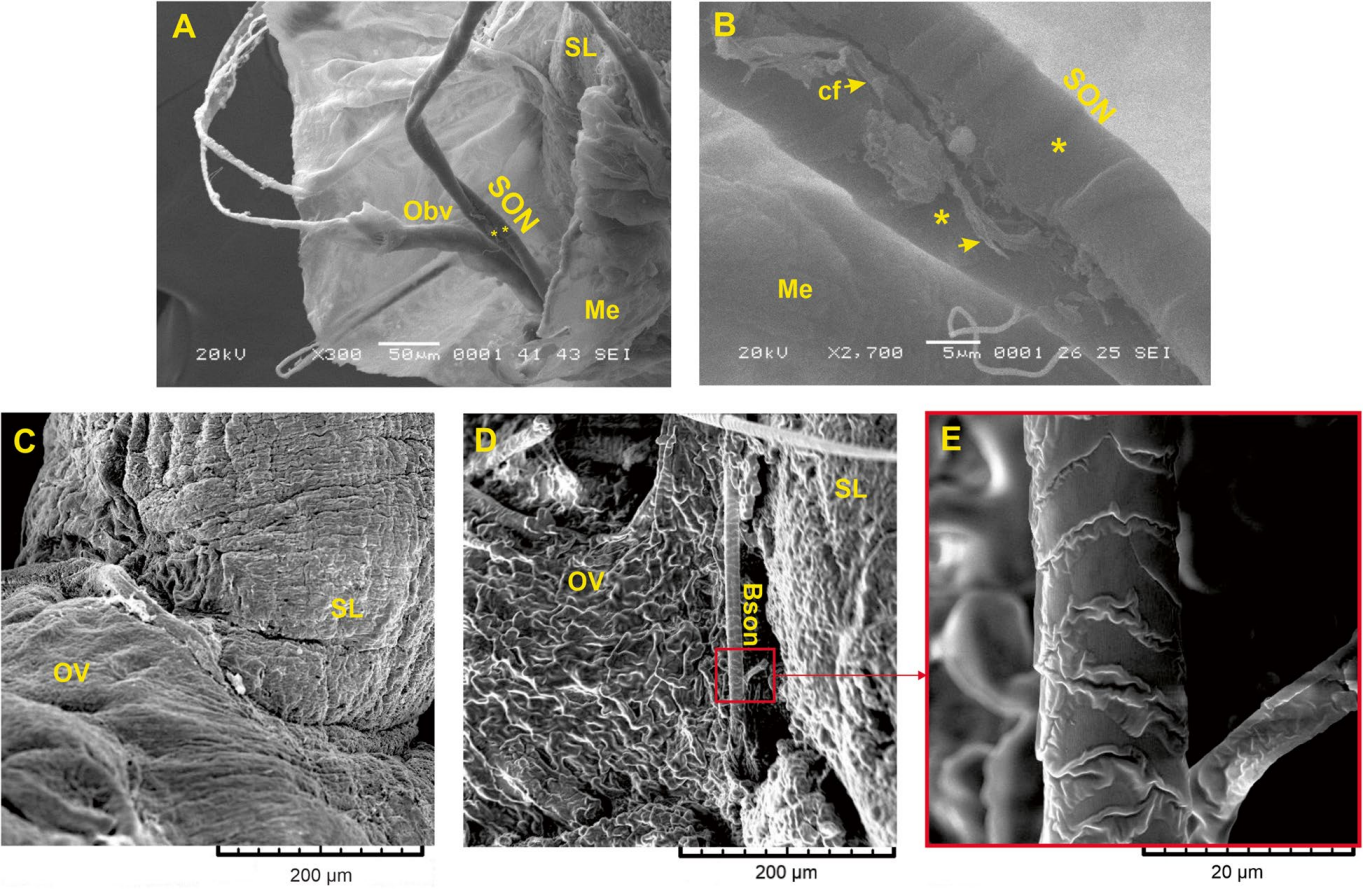
Scanning electron microscope image of the superior ovarian nerve and its path to the ovary. **A** general organization of a transverse section of the suspensory ligament. The two nerve fibers (yellow asterisks) and blood vessels are enclosed in a thin layer of mesovarium. SL, Suspensory ligament; Obv, ovarian blood vessels; SON, superior ovarian nerve; Me, mesovarium; **B** detail shows that the superior ovarian nerve is formed by two nerve fibers (yellow asterisks) with collagen fibers (yellow arrow); cf, collagen fibers; **C** detail shows the communication between the suspensory ligament and the ovary. OV, ovary; **D** a postganglionic branch of the SON, in a ventro-lateral view, where it reaches the ovary. Bson, branch of the superior ovarian nerve; E, enlargement of the red boxed region in *D*, shows the branch of the superior ovarian nerve

Along the pathway, the SON comprised two fibers, each 10 to 15 µm in diameter, that traveled together and formed coils (Fig. 4A, B). These fibers were enclosed by a thin layer of mesovarium from the suspensory ligament (Fig. 4A).

The suspensory ligament held the ovary and uterus. Figure 4C shows the insertion of the suspensory ligament into the ovary. This observation supported the notion that the SON was immersed in the gonad (Fig. 4D), however, inside the ovary, we observed only a single branch of the nerve (Fig. 4D, E).

### Neural pathway of the SON

**Fig. 5:**
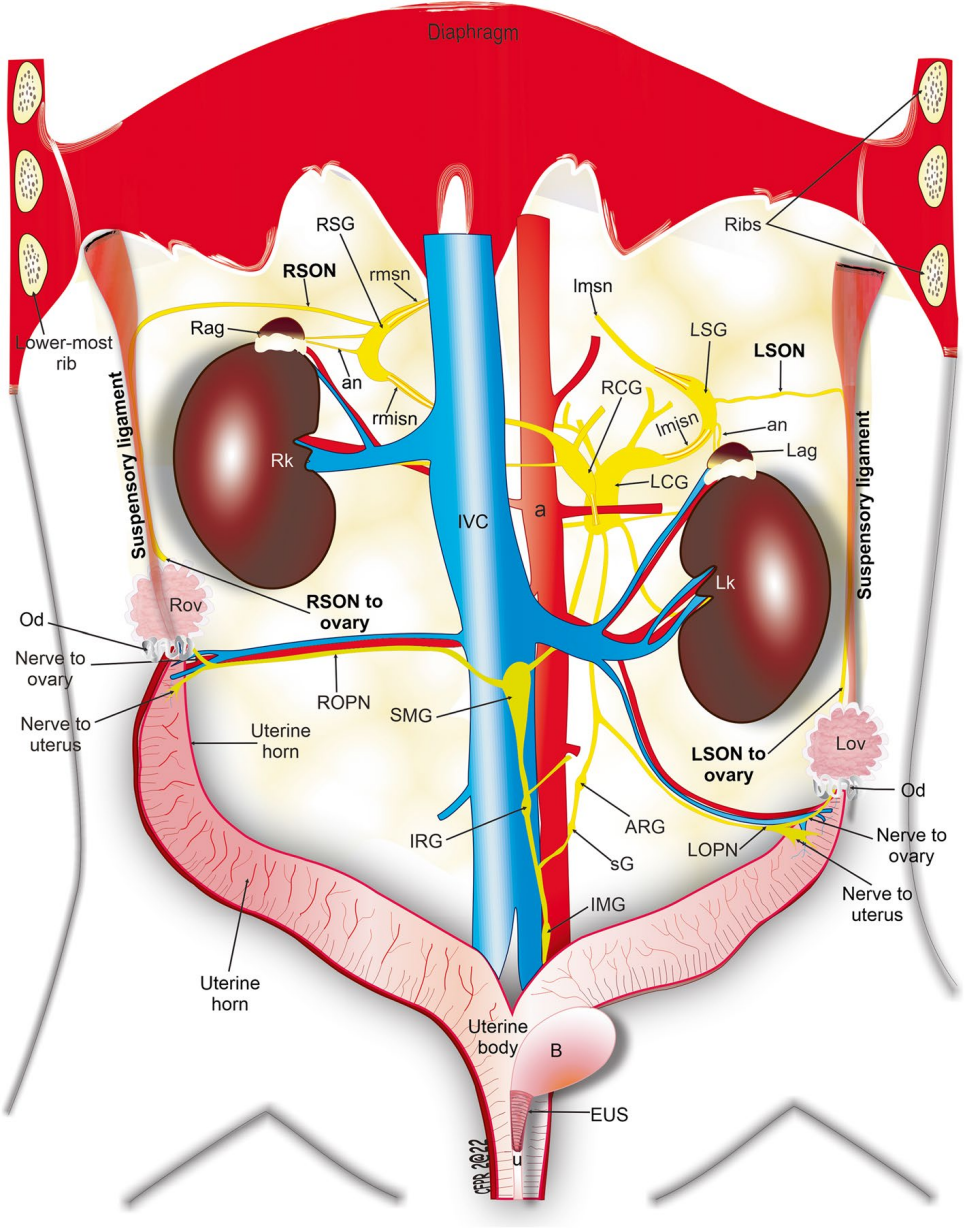
Anatomic schematic shows the major neural pathways that innervate the female rat ovaries. RSON, right superior ovarian nerve; LSON, Left superior ovarian nerve; ROPN, right ovarian plexus nerve; LOPN, left ovarian plexus nerve; RSG, right suprarenal ganglion; LSG, left suprarenal ganglion; RCG, right celiac ganglion; LCG, left celiac ganglion; SMG, superior mesenteric ganglion, IMG, inferior mesenteric ganglion; IRG, inter-renal ganglion; ARG, aorticorenal ganglion; sG, small ganglion; Rag, right adrenal gland; Lag, left adrenal gland; Rk, right kidney; Lk, left kidney; Rov, right ovary; Lov, left ovary, Od, oviduct; a, aorta; an, adrenal nerves; IVC, inferior vena cava; lmsn, left major splanchnic nerve; rmsn, right major splanchnic nerve; lmisn, left minor splanchnic nerve; rmisn, right minor splanchnic nerve; B, bladder; EUS, external urethral sphinter; u, urethra

**Fig. 6:**
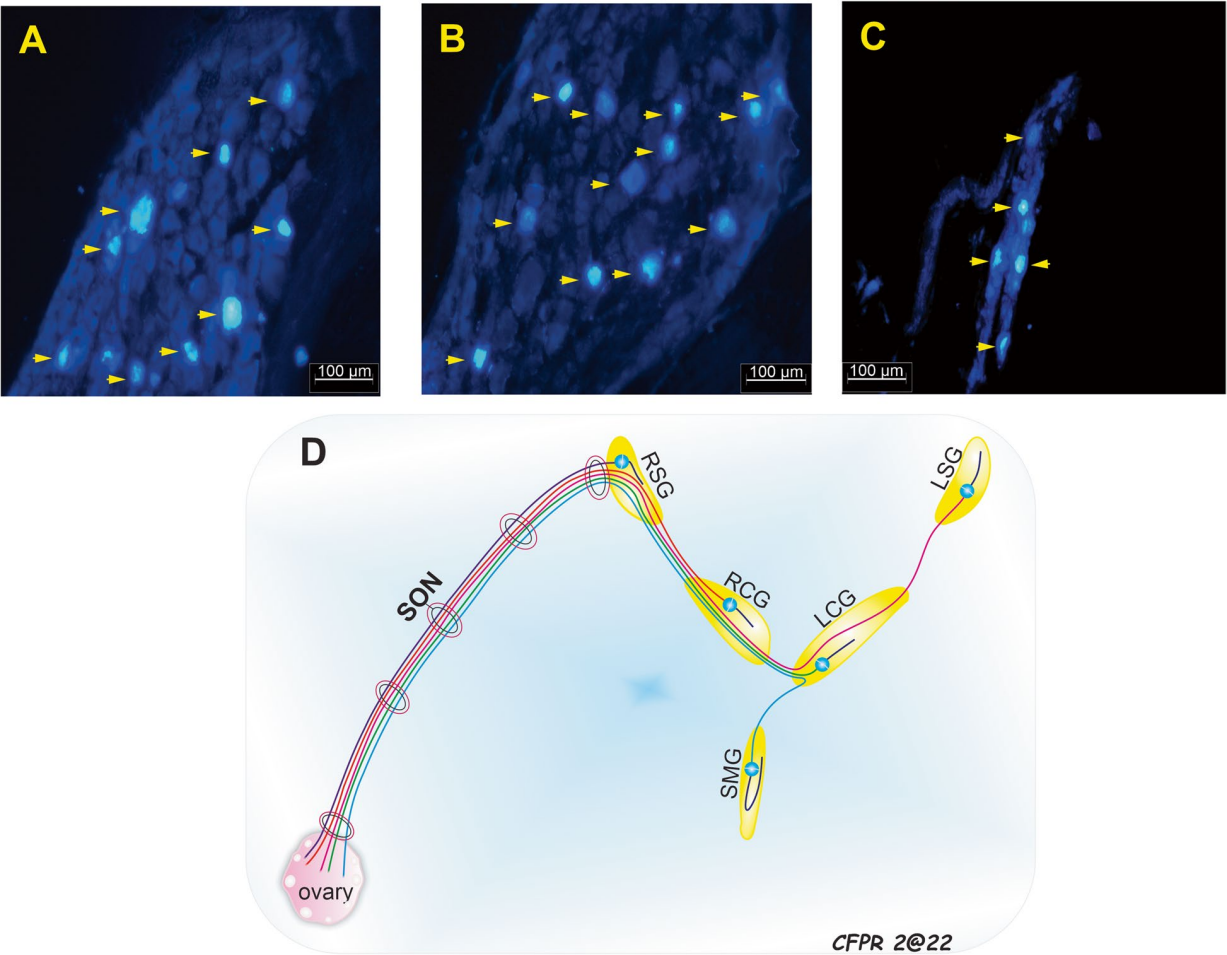
Representative photomicrographs of postganglionic neurons of the ovary (yellow arrowheads) labeled with True Blue stain in three different ganglia. Neural pathways are indicated in **A **the right suprarenal ganglion; **B** right celiac ganglion; and **C** superior mesenteric ganglion. **D **Schematic shows the superior ovarian nerve and its postganglionic neurons that project to the suprarenal ganglia, celiac ganglia, and superior mesenteric ganglion. SON, superior ovarian nerve; RSG, right suprarenal ganglion; RCG, right celiac ganglion; LSG, left suprarenal ganglion; LCG, left celiac ganglion; SMG, superior mesenteric ganglion

When TB tracer was injected into the right ovary, postganglionic somas were labeled in the right CG (101 ± 1.5 neurons; Fig. 5A), the right SG (26.3 ± 3.8 neurons; Fig. 6B), and the right SMG (10 ± 1.2 neurons; Fig. 6C). Of note, some labeled postganglionic somas were also observed in the contralateral prevertebral ganglia, i.e., the left CG and the left SG (Fig. 6D).

## Discussion

This study showed that the SG was the origin of the SON. In addition, we found that the SON was embedded in the suspensory ligament, where it continued to run, until it reached the ovary. In contrast, the OPN follows a different pathway to the ovaries [14]. Furthermore, the SON also communicates with the ovaries through the SG, the CG, and the SMG. That finding suggested that both autonomic and sensorial information from the ovaries is processed in these three ganglia [12].

Our anatomic study showed that the SON in rat emerged from the lateral border of the SG; this finding was consistent with a previous study by Baljet and Drukker [8], who observed that the SON arose from the SG. Other authors assumed that the postganglionic fibers of the SON that projected to the ovaries originated mainly from the CG [9, 12, 18]. Other studies that used neuronal tracers observed labeled postganglionic neurons in the CSMG, in a small ganglion, and in the sympathetic chain [11, 12]. It has also been described the neural ovarian communication with the central nervous system at the supraspinal level with trans-synaptic tracers, with reporting of nervous asymmetry, In this work, we corroborate the synapsis of the ovary in the first order with the CP, specifically with the SG [19, 20].

Using TB tracer we found positive neuronal staining, mainly in the CG, but also in the SG and the SMG. Additionally, we observed some positively stained neurons in the contralateral homologous ganglia, in accordance with previous findings, where a small number of neurons stained positive [11]. Consequently, our neuroanatomic study provided additional evidence that showed ovarian innervation by ipsilateral and contralateral ganglia, as demonstrated in our previous study with the OPN [21].

The role of ovarian extrinsic innervation in the physiology of the gonads has been previously researched in a in vitro model, considering the classical anatomical organization of the CG-SON-Ovary and SMG-OPN-Ovary systems [22, 23]. In the system described by Vega-Orozco [23] it was demonstrated that the SMG is closely caudal to the CG and, according to our previous work, it corresponds to a lobe of the left CG, while the SMG is attached ventrally to the vena cava [21].Thus, the results between both studies have different information, as a result of their study not including the participation of the SMG in their system SMG-OPN-Ovary on the right side, only in the CG.

On the other hand, in the study by Sosa [22], the system CG-SON-Ovary is considered different in contrast of our description, as we describe the SON anatomically attached to the SG, and no directly to the CG. For that, the anatomical arrangement showed is different. Probably, both reports [22, 23] include the right and left SG in their systems, as the two of them describe a similar trajectory of the SON like we showed, and both mentioning small ganglia around the CG. Nevertheless, we agree that the CG is the principal center of the autonomic nervous system where converge the neural information from the ovary through the SON.

The postganglionic fibers that make up the SON travel bilaterally, starting from the CP, then running along the minor splenic nerve to reach the SG, where they emerge to join the suspensory ligament and innervate the ovary (Figs. 1 and 2). Once the SON is submerged in the two intertwined branches can be observed; however, only one branch enters the ovary; the characteristics of this nerve were not described previously (Fig. 4B). Therefore, we suggest that the second branch of the SON continues caudally, to innervate caudal structures, such as the oviduct and the cranial uterus [24].

These findings provided an opportunity to determine whether the neuronal process of each prevertebral ganglion followed a direct pathway to the target organ or whether there was communication between first-order neurons (neuron-neuron), which would indicate a ganglionic interaction between ipsilateral and contralateral plexus ganglia (Fig. 6D). This relationship could explain the differences in physiological responses of the ovaries (synthesis of steroid hormones by the ovary, ovulation, follicular development and asymmetry) [25–31] when performing unilateral resections of the SON, OPN, or vagus nerve [7, 32].

In studies with SON axotomies, where the suspensory ligament is cut, the effect of the severed nerve has been unclear, because the SON participates in the regulation of the blood vessels, which are innervated by adrenergic fibers [9, 15, 33]. Therefore, by injuring the suspensory ligament during SON denervation, the hemodynamic events in the ligament can be interrupted, which would affect the ovaries; in addition, the anatomic and supportive link with the uterus would be lost. Accordingly, our results highlighted the importance of maintaining an intact suspensory ligament and blood vessels, to avoid affecting ovarian functions.

The SON is a very thin nerve composed mainly of unmyelinated fibers [9]. It can be located anatomically at the points where it enters or leaves the suspensory ligament and at the point just before it enters the ovary. In these areas, our results were not consistent with those reported in previous studies [1, 9]. Consequently, we propose that the ideal location for performing a SON denervation is the cranial section, which is attached to the adrenal ganglion; specifically, the part of the SON that is located between the lower-most rib and the lateral edges of the adrenal gland.

## Conclusions

This study showed that the postganglionic fibers that compose the SON originated bilaterally in the prevertebral ganglia, mainly in the SG. However, these preganglionic fibers also had relationships with the CG and SMG. From the SG, the nerve traveled alone for a short distance, and then inserted into the suspensory ligament, where it projected caudally to the ovary, the oviduct, and the cranial part of the uterus. Moreover, we found that the SON comprised two branches that were separated, just before entering the ovary.

The pathway that the SON took to the ovaries was complementary to the pathway traveled by the OPN, and the information of both nerves is processed in prevertebral ganglia. These findings may unify a great variety of previous anatomical descriptions about the origin, trajectory, and the distribution of SON fibers that innervate the ovary in the rat.

## Data Availability

The datasets supporting the conclusions of this article are included within the article.

## Abbreviations

SON: Superior ovarian nerve
OPN: Ovarian plexus nerve
CG: Celiac ganglion
SMG: Superior mesenteric ganglion
SG: Suprarenal ganglion
CSMG: Celiac superior mesenteric ganglia complex
CP: Celiac plexus
TB: True blue
SEM: Scanning electron microscopy
PBS: Phosphate buffer
